# The Non-conventional Effects of Hypovitaminosis D: A Pandemic Even in Sunlight-Rich Countries

**DOI:** 10.7759/cureus.59267

**Published:** 2024-04-29

**Authors:** Ghania Qureshi, Madjda Khemissa, Ganna Amr, Raghavendra Bhat

**Affiliations:** 1 Internal Medicine, Ras al Khaimah Medical and Health Sciences University, Ras Al Khaimah, ARE; 2 Internal Medicine, Ras al Khaimah Medical and Health Sciences University, Ras Al Khaima, ARE

**Keywords:** public health, extraskeletal manifestations, metabolic bone diseases, vitamin d deficiency, hypovitaminosis d

## Abstract

The synthesis and absorption of Vitamin D play crucial roles in numerous bodily functions, yet deficiencies persist due to factors like insufficient sunlight exposure and dietary inadequacy. Research underscores the significance of lifestyle elements such as diet, sun exposure, and physical activity in maintaining optimal Vitamin D levels. Strategies aimed at tackling deficiencies emphasize supplementation alongside lifestyle adjustments, especially in regions with abundant sunlight like the Middle East and North Africa (MENA).

Despite the abundance of sunshine in the Arab world, there remains a prevalent issue of Vitamin D deficiency. This problem arises from various factors, including cultural practices such as traditional clothing covering most skin areas, which limit sun exposure, and environmental factors like air pollution that reduce UV penetration. Dietary habits and lifestyle choices also contribute to this deficiency.

Dealing with the ongoing pandemic requires a focused effort to enhance awareness. While some individuals may recognize common diseases caused by Vitamin D deficiency, such as rickets and osteomalacia, many remain unaware of the broader health risks associated with the condition, including non-skeletal manifestations. Additionally, there is a lack of understanding regarding the numerous hidden benefits of this hormone. Therefore, prioritizing educational initiatives that delve into these aspects is essential to effectively combat the current health crisis.

This literature review aims to report both skeletal and extraskeletal consequences of hypovitaminosis and briefly discuss the cause of paradoxical vitamin D deficiency in sunny regions like the MENA.

This was done by reviewing pertinent articles published between January 2000 and January 2024, sourced from databases such as PubMed, UpToDate, Scopus, and CINAHL, focusing exclusively on English language literature and using keywords such as “Vitamin D deficiency” and “Extraskeletal manifestations.”

## Introduction and background

**Figure 1:**
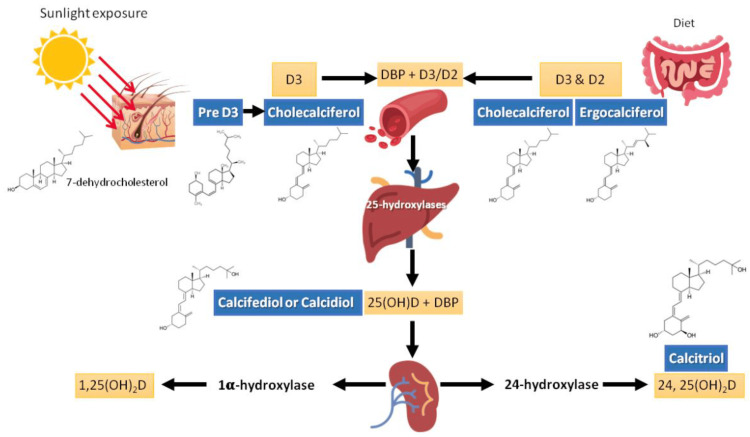
Vitamin D synthesis Pre D3: pre-vitamin D3; DBP:vitamin D-binding protein; 25[OH]D: 25-hydroxycholecalciferol; 1,25(OH)_2_D: 1,25-dihydroxyvitamin D

Vitamin D (calciferol) is a fat-soluble vitamin that is naturally present in certain foods (fatty fish, liver, red meat, egg yolks, fortified dairy, and grain products) and is endogenously produced when ultraviolet (UV) rays from sunlight strike the skin and trigger its synthesis. Exposure to sunlight allows UV radiation to penetrate the epidermis and photolyze provitamin D3 to previtamin D3. Then, pre-vitamin D3 can be isomerized to vitamin D3. Once formed, vitamin D enters the circulation and is metabolized first to 25 hydroxyvitamin D (25(OH)D) in the liver, then to the hormonal/active form 1,25-dihydroxyvitamin D (calcitriol) in the kidneys (Figure 1) [1-3].

Vitamin D has numerous functions in the human body such as promoting calcium absorption in the gut, maintaining adequate serum calcium and phosphate concentrations, bone growth and remodeling, reducing inflammation, as well as modulation processes such as cell growth, neuromuscular and immune function, and glucose metabolism [4].

Risk factors

Vitamin D deficiency has been linked to multiple risk factors such as the absence of direct sunlight, old age, a diet lacking vitamin D-rich food, diseases that may hinder the body from absorbing vitamin D (for example, celiac disease), use of certain medications like anti-epileptic drugs, etc. [5,6].

Individuals belonging to countries that have low amounts of sunlight are more likely to suffer from vitamin D deficiency than those from sunlight-rich countries [7]. However, a study done by Chakhtoura et al. demonstrated a high prevalence of vitamin D deficiency in the Middle East and North Africa (MENA) region which is renowned to be a group of countries with ample amounts of sunlight throughout the year. This phenomenon has been linked by researchers to reduced sunlight exposure, with individuals choosing to remain indoors to avoid extreme heat. Other factors contributing to this pandemic include dark skin tones and cultural dress codes in regions like the Arab world, where clothing covers much of the body's surface area. Moreover, a deficiency in a diet rich in vitamin D is a significant contributing factor [8].

‌In 2022, a study was performed by Al Zarooni et al. in Abu Dhabi where a total of 700 people were randomly chosen from the 12,346 participants who showed up for the Weqaya population comprehensive cardiovascular risk factor screening. Out of these, 400 completed a questionnaire asking about their dietary intake, sun exposure, clothing, physical activity, and symptoms of vitamin D deficiency. It was evident that participants who ate more cod liver oil, tuna, salmon, and eggs, wore garments that exposed their arms and legs and engaged in regular physical exercise had higher vitamin D levels [9].

Apart from supplementation, lifestyle modifications are crucial to treat vitamin D deficiency. Outdoor activities between 10 am-3 pm may serve as a surrogate factor for exposure to ultraviolet radiation. In countries with frequent sun exposure such as the United Arab Emirates, residents are advised to spend about 30 minutes under the sun. For those with lighter skin that burns more easily, 15 minutes should suffice [10].

Hypovitaminosis D: diagnosis and epidemiology

The diagnosis of hypovitaminosis D is based on a low 25-hydroxyvitamin D3 level. The normal range of 25-hydroxy vitamin D is measured in nanograms per milliliter (ng/mL). A level between 30 and 50 ng/mL is recommended by experts. An adult with a serum 25-hydroxyvitamin D level of 20 to 30 ng per mL is said to have vitamin D insufficiency, and a level less than 20 ng per ml is known as deficiency [11].

Research has shown that about 1 billion people around the globe suffer from Vitamin D deficiency, while 50% of the population suffers from vitamin D insufficiency [12]. About 30%-50% of children and adults in the United Arab Emirates (UAE), Australia, Turkey, India, and Lebanon have 25-hydroxyvitamin D levels less than 20 ng/m [13]. The consequences of this can be catastrophic. Classically, vitamin D deficiency is associated with metabolic bone diseases like rickets in children and osteomalacia in adults. However, recent research has linked vitamin D levels with several other health issues: Type 2 diabetes, eclampsia, depression, HIV infection, COPD, acne, hyperparathyroidism, etc. [14,15].

Investigations and reliable tests used to diagnose vitamin D deficiency**

Vitamin D status can be determined using radioimmunoassay, competitive protein-binding assays, high-pressure liquid chromatography, and liquid chromatography-tandem mass spectrometry (LC-MS). LC-MS is the gold standard technology for vitamin D testing. However, measurement of serum 25(OH) D levels is frequently performed using immunoassays [16]. Ingested and cutaneous produced vitamin D is converted to 25-hydroxyvitamin D. In serum only a fraction of that 25(OH) D is converted to its active metabolite 1, 25(OH)2D. Therefore, measurement of the total 25(OH) D level is a very accurate laboratory test used to assess vitamin D status in the body [17,18].

## Review

Metabolic bone diseases

Rickets

Extreme and prolonged vitamin D deficiency may lead to rickets in childhood. Rickets is a disease characterized by bowed legs, short stature, and widening of the epiphysis. The patient may present with delayed growth, delayed motor skills, bone pains, or generalized muscle weakness. Taking proper history, performing physical examination, use of radiograph images, and biochemical testing are necessary to arrive at a diagnosis [19].

This illness can be classified in various ways, with one particularly relevant classification being calciopenic rickets. This type arises from a lack of vitamin D nutritionally, and less commonly due to disruptions in its metabolism and cellular functions [20]. Preventing nutritional rickets involves a combination of sunlight exposure, vitamin D and calcium supplements, or enriched dietary sources. If left untreated, rickets can result in stunted growth, abnormal spinal curvature, bone malformations, dental issues, seizures, and so forth [20].

Osteomalacia

In adults, vitamin D deficiency may lead to osteomalacia. This is a generalized bone disorder characterized by impairment of mineralization, leading to the accumulation of an unmineralized matrix or osteoid in the skeleton. In other words, it is the “softening” of bones. Patients usually present with weakened muscles, bone disfigurement, and pain in the bones and muscles. This condition can be detected with the use of blood tests, x-rays, bone density tests, and in some cases, a bone biopsy [21]. Treatment of osteomalacia includes consuming more vitamin D-rich food such as fish, egg yolks, cow's milk, etc. [22]. Taking vitamin D or phosphate supplements, and lifestyle changes like daily sunlight exposure, limiting consumption of alcohol, and exercise can play a significant role in the promotion of strong and healthy bones [23].

Autoimmune

With significant effects on maintaining health, the development of diseases, and notably autoimmune illnesses, vitamin D plays a crucial part in regulating immune function. Low blood levels of 25(OH) D have been linked to a higher risk of developing autoimmune diseases and/or having active disease states [24].

1,25-dihydroxyvitamin D binds to the receptor of vitamin D to affect both innate and adaptive immunity in several ways, particularly by reducing inflammation and promoting tolerogenic responses. In the case of rheumatoid arthritis, an autoimmune disease, it has been shown that vitamin D can inhibit local inflammation by lowering the expression of aromatase in human macrophages. Thus, a vitamin D deficiency may be a factor in the inflammation of the synovium in RA [24].

In animal studies, vitamin D has demonstrated a favorable effect on the onset and course of SLE. Specifically, 1 alpha,25-dihydroxyvitamin D3 (1,25(OH)2 D3) increases Treg cells, decreases Th1, Th2, Th17 cells, and autoantibody production, hence reducing the symptoms of disease [24].

Additionally, a literature review by Liu et al. suggested that 1,25-(OH)2D3/vitamin D receptor (VDR) may be a good target for Systemic lupus erythematosus (SLE) therapy since it upregulated p27 expression while down-regulating S-phase kinase-associated protein 2 (Skp2) expression during SLE recovery [25].

According to a study conducted by Murdaca et al., numerous autoimmune illnesses (SLE, thyrotoxicosis, type 1 diabetes, multiple sclerosis (MS), iridocyclitis, Crohn's disease, ulcerative colitis, psoriasis vulgaris, seropositive rheumatoid arthritis, and polymyalgia rheumatica) were shown to have an inverse relationship with vitamin D [26].

Type 2 Diabetes

One of the numerous benefits of vitamin D is its ability to enhance the body’s sensitivity to insulin, and consequently decrease the risk of insulin resistance which is known to be a precursor to type 2 diabetes [27].

The findings of vitamin D receptors in pancreatic β cells as well as musculoskeletal and adipose tissue also support the theory that vitamin D influences insulin synthesis and secretion and that a deficiency can affect the capacity of β cells to convert proinsulin into insulin [28,29].

In addition, it has been shown that patients with decreased plasma vitamin D concentrations have characteristics of metabolic syndrome: elevated serum concentrations of glucose, total cholesterol, low-density lipoproteins, triglycerides, glycosylated hemoglobin, and a high body mass index [30].

The best-known role of 25-hydroxyvitamin D is its ability to maintain homeostasis by enhancing calcium and phosphorus uptake in the gut. Research has demonstrated that changes in the levels of these vitamins can play a role in the onset of diabetes [31].

Additionally, fluctuations in pre-meal glycemic control and HbA1c levels have been noticed among individuals with type 2 diabetes, with levels tending to be higher during the winter months. This pattern aligns with reduced sunlight exposure and limited skin exposure to vitamin D during winter, indicating a potential connection between seasonal changes in glucose levels and variations in vitamin D levels [32].

Pre-eclampsia

Pre-eclampsia is a known obstetric disease, affecting multiple body systems, characterized by high blood pressure and proteinuria after the 20th week of pregnancy. Maternal diet is one of the most important factors related to its etiology. A diet that is insufficient in calcium, magnesium, vitamins D and C is a major contributing factor [33].

Accumulating evidence has linked vitamin D deficiency to pre-eclampsia. Increasing vitamin D levels may improve the invasion of human extravillous trophoblasts needed for normal placentation [34]. The active form of vitamin D 1,25-dihydroxy vitamin D3 was shown to regulate the function of genes associated with normal implantation, placental invasion, and angiogenesis. Furthermore, due to its effect on angiogenesis, vitamin D can prevent proteinuria by acting on angiogenic processes through effects on vascular endothelial growth factor (VEGF) gene transcription [35].

**Figure 2:**
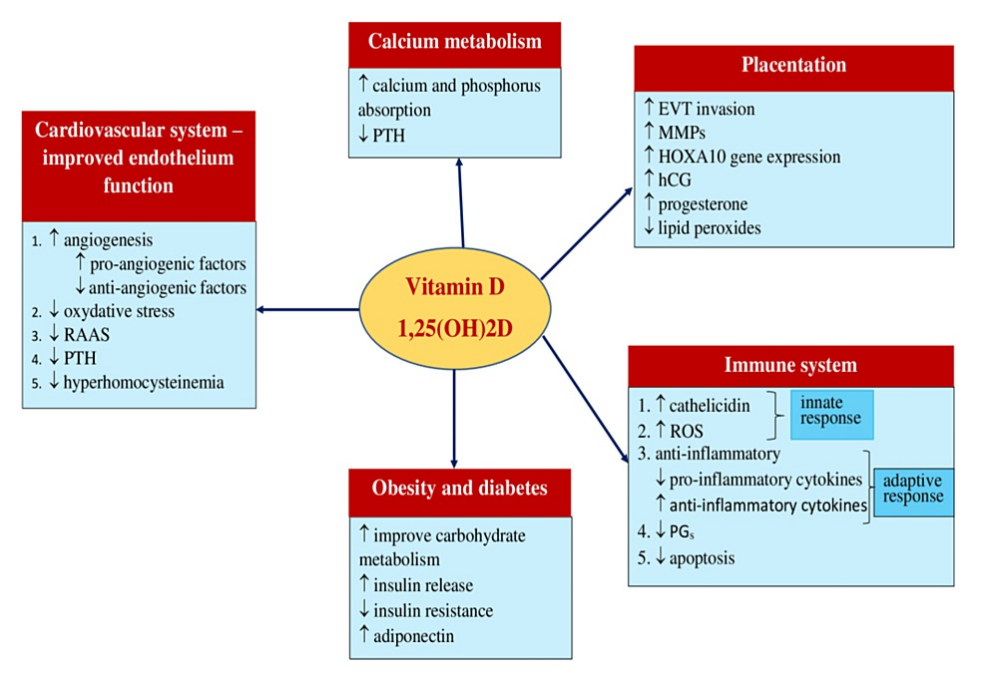
Use of vitamin D in prevention of pre-eclampsia [35] 1,25(OH)2D: 1,25-DihydroxyvitaminD; RAAS: Renin-Angiotensin-Aldosterone-System; PTH: Parathyroid Hormone; EVT: Extravillous Trophoblast; MMPs: Matrix metalloproteinase; HOXA10: Homebox A10/a10; hCG: Human chorionic gonadotropin; ROS: Reactive Oxygen Species; PG: prostaglandin

The immunomodulatory properties and vascular function of vitamin D are also hypothesized to reduce the risk of preeclampsia development as vitamin D inhibits Th1 upregulation, which is seen in preeclampsia placentas. Moreover, decreased expression of pro-inflammatory cytokines such as TNF-α and IL-6 in placental tissues was seen when treatment with vitamin D was supplied to patients with preeclampsia compared to trophoblast cell cultures that did not receive that treatment. A randomized controlled clinical trial that aimed to determine the effect of vitamin D supplements on reducing the chances of recurrent preeclampsia demonstrated that maternal vitamin D deficiency may increase the inflammatory reaction and risk of hypertension (Figure 2) [35,36].

Moreover, in a cohort study performed on 13,806 pregnant women, maternal vitamin D deficiency at 23-28 weeks of gestation was strongly associated with an increased risk for severe pre-eclampsia [37].

Depression

The precise mechanism of the biological relationship between vitamin D and depression is unclear. However, there are potential mechanisms such as the imbalance between the excitatory neurotransmitter glutamate and inhibitory neurotransmitter Gamma-aminobutyric acid (GABA) caused by vitamin D deficiency. This is known to have an impact on cell signaling. By controlling intracellular calcium storage and cellular signaling, vitamin D may have a possible role in reversing this calcium and neurotransmitter imbalance [38].

Additionally, vitamin D influences the hypothalamic-pituitary-adrenal axis, which controls the adrenal cortex's synthesis of the monoamine neurotransmitters adrenaline, norepinephrine, and dopamine. It also guards against dopamine and serotonin shortage [38].

It is biologically possible for vitamin D to be linked to brain activity due to the presence of vitamin D receptors in the brain and its critical role in immune and nervous system responses [39,40]. A cross-sectional study done by Croll et al. on 2,716 adults in the Netherlands revealed that individuals with vitamin D insufficiency had smaller hippocampus (one of the areas linked with depression) and less brain tissue overall [41].

**Figure 3:**
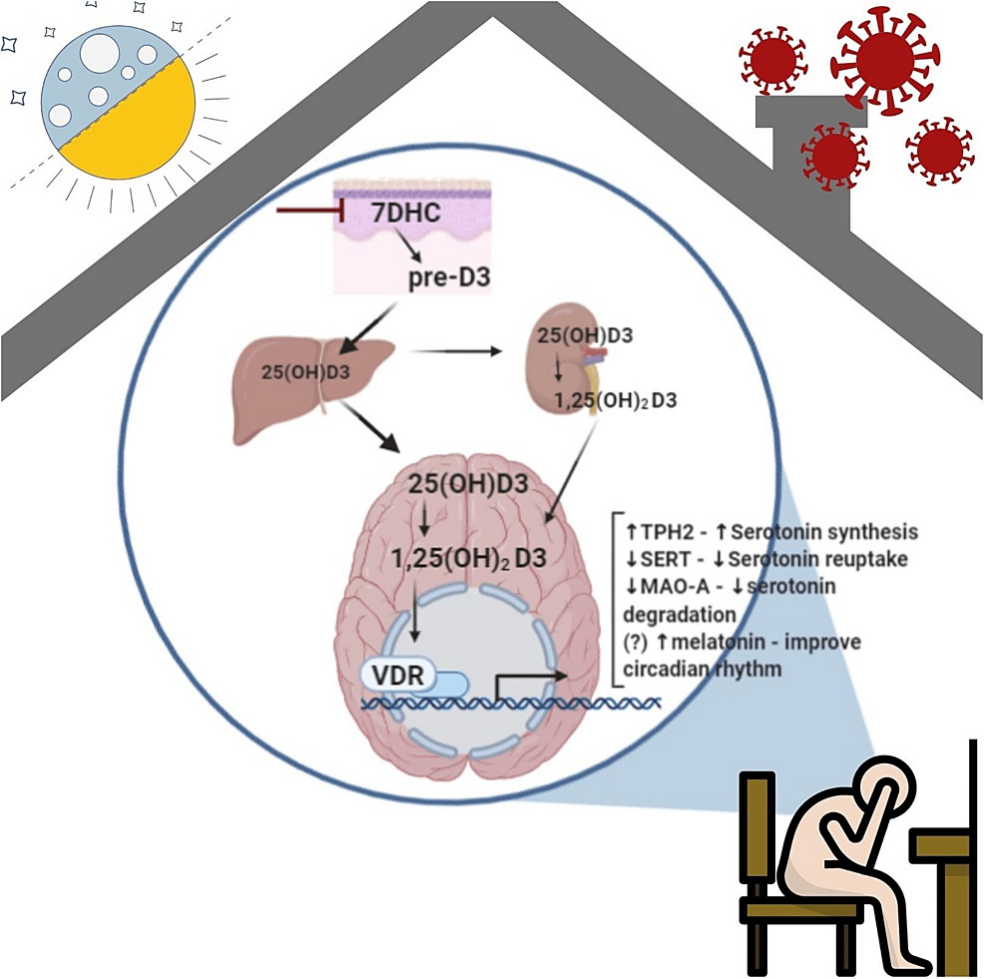
Role of vitamin D in the pathogenesis of depression 7DHC: 7-dehydrocholesterol; 25OHD3: 25-hydroxyvitaminD3; 1,25(OH)D3: 1,25-dihydroxyvitaminD3; VDR: Vitamin D receptor; TPH: Tryptophan hydroxylase; SERT: Serotonin Reuptake Transporter; MAOA: Monoamine Oxidase A

A growing body of research has shown that vitamin D is a powerful regulator of the production of neurotrophic factors such as neurotrophin (NT)-3, brain-derived neurotrophic factor (BDNF), and nerve growth factor (NGF). The survival, development, and migration of neurons all depend on neurotrophic substances [42]. The expression of tryptophan hydroxylase 2 (TPH2), serotonin reuptake transporter (SERT), and monoamine oxidase-A (MAO-A), the enzyme responsible for serotonin catabolism, are all affected by VDR reception of signals from 1,25-dihydroxyvitamin D3 (Figure 3) [43].

Various studies demonstrate a connection between depression and vitamin D levels. A prospective cohort study performed by Jorde et al. mentioned that there is an inverse connection between depression and 25(OH) D levels. The Beck Depression Inventory-II (BDI-II) was used to assess depression in the 3,369 men who were participating in the European Male Aging Study and radioimmunoassay was used to measure blood 25(OH) D. Results demonstrated that higher BDI-II scores were related to lower levels of 25(OH)D [44,45].

Another study conducted by Hoogendijk et al. using data from The Longitudinal Aging Study Amsterdam showed that depressive participants had vitamin D levels 14% less compared to non-depressive participants out of 1,282 people. This difference was determined using the Centre for Epidemiologic Studies Depression scale [46].

Finally, a study done by Ganji et al., including 7,970 non-institutionalized U.S. citizens between the ages of 15 and 39, showed that individuals who have serum vitamin D levels below 50 nmol/L have a markedly higher risk of developing depression than those whose levels are above or equal to 75 nmol/L [47].

Previous researchers have also raised the idea of reverse causation. As social isolation is a sign of depression, individuals spend fewer hours outdoors in the sun. Additionally, they often struggle to care for themselves. It is possible that they do not eat enough nutritious food or take their required supplements. Their behavior and mental processes are altered by depression, which leads individuals to act in ways that may exacerbate vitamin D deficiency [48].

The literature already has enough evidence to recommend screening for and treating vitamin D deficiency in individuals with depression, as it is simple, affordable, and may improve depression outcomes. However, several problems in the connection between depressive symptoms and low levels of vitamin D remain questionable and require further research [49,50].

Multiple Sclerosis

MS is a demyelinating, inflammatory condition, which involves the central nervous system, affecting up to 2.8 million persons globally. Clinically, this condition is characterized by progressive, relapsing neurological impairment [51]. Pathologically, MS is characterized by focal plaques of demyelination with activated microglia and abundant peripheral inflammatory cells in the CNS. Since vitamin D was known to have immunoregulatory properties, it was found that low vitamin D resulted in defective immune responses and hence the development of MS. However, vitamin D receptors are present in many cell types, including neurons, oligodendrocytes, astrocytes, and microglia. This leads to the possibility that low vitamin D may alter the CNS in a manner that makes it vulnerable to inflammation and the development of MS [51].

Hypovitaminosis D has long been thought of as a risk factor for MS. It is currently one of the most studied factors in terms of new clinical therapeutic implications [52]. Vitamin D regulates a number of MS risk genes, the strongest being the variation of the HLA-DRB1 gene. The promoter of this variation has a functional vitamin D response element. Myelin-specific autoreactive T cells may be able to evade negative selection, which leads to the later development of MS if vitamin D insufficiency and down-regulated expression of HLA-DRB1*15 coexist. Vitamin D can also target a number of other MS risk genes, like IL2RA, which encodes CD25, a subunit of the high-affinity IL-2 receptor present on effector T cells [53].

Multiple studies have demonstrated a decreased risk of MS in persons who consume greater vitamin D due to its anti-inflammatory and immunomodulatory effects. One such example is the two-year-long randomized clinical trial CHOLINE, where interferon beta-1a-treated MS patients received 100,000 IU of oral cholecalciferol or a placebo twice a week. Positive imaging improvements were observed and the therapy group’s average EDSS (expanded disability status scale) score was significantly lower, despite the fact that the intended outcome (change in annualized relapse rate) was not accomplished [54]. Finally, a study conducted by Islam et al. involving discordant monozygotic twins with MS, showed that the twin with MS reported much less sun exposure as children than their unaffected sibling [55].

Chronic Obstructive Pulmonary Disease

Chronic obstructive pulmonary disease (COPD) is a progressive disease characterized by restricted airflow and breathing difficulty [56]. The risk, intensity, and aggravation of COPD were negatively correlated with serum vitamin D levels [57,58]. The Vitamin D Council, USA claims that vitamin D concentrations over 30 to 40 ng/mL may lower the incidence of COPD [59].

Vitamin D is known to improve immunological responses to respiratory infections, which frequently cause COPD episodes. It lessens damaging inflammatory reactions, speeding up healing and minimizing injury to the lung’s structural integrity [60]. Emerging data suggest that host defenses toward respiratory tract infections depend in part on vitamin D-mediated innate immunity, specifically through increased production of the human cathelicidin antimicrobial peptide (hCAP-18) [61].

Vitamin D may also lessen the IL-6 response brought on by oxidative stress and particulate matter. The main factor that causes airway irritation is smoking. Cigarette smoke components prevent VDR translocation in human alveolar epithelial cells and produce a regional vitamin D signaling downregulation, which makes it difficult for COPD patients to manage pro-inflammatory processes in their airways. This suggests that the symptoms of COPD patients may be lessened by providing them with vitamin D supplements [62].

When considered collectively, several lines of research point to a possible function of supplemental vitamin D in reducing flare-ups of COPD [63]. Additionally, COPD progression is shown by forced expiratory volume (FEV1) decline, and a decreased FEV1 is associated with low blood 25-hydroxyvitamin D levels, which is found in 60% to 75% of individuals with severe COPD [59].

A randomized, double-blind, placebo-controlled experiment was conducted by Martineau et al. in 60 general practices and four Acute National Health Service Trust clinics in London, UK, for persons with COPD. Two hundred and forty patients were divided into two groups at random: 122 participants in the vitamin D3 group and 118 in the placebo group. In COPD patients having an average of vitamin D levels less than 50 nmol/L, vitamin D3 administration protects against moderate or severe exacerbation [64].

Acne

Acne is a multifactorial condition; Growth and differentiation of keratinocytes and sebocytes, are influenced by vitamin D. Comedo formation is likely the initial stage in the development of acne and is caused by changes in the pattern of keratinization inside the pilosebaceous follicles [65]. Vitamin D possesses anti-comedogenic and also antioxidant effects. Consequently, its deficit may promote the development of acne [66].

Moreover, by stimulating antimicrobial peptides such as LL-37 in human sebocytes, vitamin D exerts antimicrobial properties [67]. The target cells for physiologically active vitamin D metabolites are human sebocytes and keratinocytes, which are reached by nuclear VDRs [68]. It has been noted that vitamin D3 inhibits keratinocyte proliferation and promotes keratinocyte differentiation [66]. This is explained by the VDR-mediated genetic effect that causes an arrest of sebocytes in the G1 phase, which in turn prevents cell proliferation [69]. Therefore, finding vitamin D receptors in human sebocytes and evidence that vitamin D influences lipid and cytokine formation point to a potential link between vitamin D and the pathogenesis of acne [70].

There are several biochemical processes via which vitamin D possesses anti-inflammatory actions. This was demonstrated by adding vitamin D in cultured sebocytes in which there was a decrease in the production of inflammatory indicators such as IL-6, IL-8, and matrix metalloproteinase 9 [71]. Additionally, there is evidence that vitamin D prevents P. acnes from causing Th17 differentiation, which results in lower levels of the inflammatory cytokine IL-17 [72].

A study by Rasti et al. was conducted on 43 individuals with recently diagnosed nodulocystic acne and 46 healthy control subjects. When compared to the participants in the control group, individuals with nodulocystic acne had serum vitamin D levels that were quite low [66].

*Diabetic Retinopathy and Glaucoma* 

From impaired tear function to diabetic retinopathy, a lack of vitamin D has been associated with multiple ocular pathologies. A possible explanation for the association between lower vitamin D levels and glaucoma is the mechanism of impaired ocular blood flow. Studies have reported that vitamin D regulates the renin-angiotensin system and improves endothelial cell-dependent vasodilation, which affects peripheral and microvessel circulation [73].

Research has indicated that administering vitamin D can decrease the retinal presence of VEGF, potentially offering protective benefits to the retina. Furthermore, vitamin D is found to have a correlation with decreasing intraocular pressure, which is the main target for glaucoma treatment [74]. It has been shown that an imbalanced immune system is a contributor to neurodegenerative diseases of the optic nerve. Since vitamin D plays a role in the regulation of immune cell functions, this effect may be protective of the optic nerve [74]. Vitamin D is known to activate calcium channels, a mechanism that helps regulate oxidative stress in neurons, which is important in glaucomatous optic nerve damage [75].

Hyperparathyroidism

Hyperparathyroidism is a disease characterized by excess activity in one or more parathyroid glands. The clinical severity of individuals diagnosed with primary hyperparathyroidism is more severe in those with concomitant vitamin D deficiency. Additionally, vitamin D deficiency and insufficiency seem to be more prevalent in patients with primary hyperparathyroidism than in geographically matched populations. The association between vitamin D deficiency and primary hyperparathyroidism has clear implications [76].

Studies found an increased incidence of vitamin D insufficiency in moderate primary hyperparathyroidism. While there was no link between 25(OH)D levels and adenoma weight, they did observe that low levels of this type of vitamin D were related to signs of higher disease severity [76].

Vitamin D deficiency is also an under-recognized but increasingly important cause of secondary hyperparathyroidism. Levels of 25-hydroxyvitamin D3 less than 20 ng per mL trigger a compensatory increase in parathyroid hormone (PTH) and, hence, accelerate bone resorption. This suggests that vitamin D deficiency occurs before the lower limits of traditional population-based values for PTH. Unlike primary hyperparathyroidism, PTH levels are usually less than 100 pg per mL in secondary hyperparathyroidism caused by hypovitaminosis [77].

Cancer

According to epidemiological research, vitamin D levels and the risk of breast cancer are inversely related. Low vitamin D consumption has also been linked to the development of colorectal cancer. Studies have linked conditions like multiple myeloma, ovarian cancer, and prostate cancer to vitamin D deficiency [78].

Hofmann et al. investigated within-person variability in 25(OH)D concentrations in blood samples obtained at three-time points over a five-year period from 29 individuals in the prostate, lung, colorectal, and ovarian cancer screening trial. They found relatively low within-subject variability and rather good correlations in 25(OH)D assessed from samples obtained at the study's baseline, one-year, and five-year marks [79].

As the brain, prostate, breast, and colon all have VDR, one of the various biological effects of 1,25(OH)2D binding to VDR is the stimulation of differentiation and apoptosis as well as the suppression of proliferation, angiogenesis, and metastatic potential. As a result, vitamin D is thought to be crucial for the prevention and treatment of cancer [80].

Vitamin D deficiency treatment toxicity

The commonly known treatment of hypovitaminosis D is vitamin D supplementation. The available forms of supplements include ergocalciferol (vitamin D2) and cholecalciferol (vitamin D3). As individuals have become more aware of vitamin D deficiency and bone-related problems, the use of vitamin D supplementation has increased drastically over the past few years and hence vitamin D has become a popular supplement. However, excess use of vitamin D supplementation without medical monitoring might result in vitamin D intoxication. Clinical signs of hypervitaminosis D include confusion, apathy, recurrent vomiting, abdominal pain, polyuria, polydipsia, and dehydration [81]. Serum 25-hydroxyvitamin D concentrations greater than 150 ng/mL (375 nmol/L) are the distinctive features of hypervitaminosis D due to supplementation [81].

## Conclusions

Overall, this review aims to highlight the importance of expanding the current knowledge on the effects of hypovitaminosis D beyond metabolic bone diseases. A lack of vitamin D, along with confounding factors, has been linked to chronic inflammation, heart disease, brain health, frailty in old age, and even hair loss. It is important to note that Vitamin D deficiency is an ongoing pandemic with no end in sight. This is mainly due to a lack of awareness. People need to be informed of the severe outcomes of hypovitaminosis D and realize the numerous silent benefits of the hormone as it leads to the promotion of immune health and bone health, management of blood sugar levels, facilitation of hormone regulation, protection against cancer, improvement in mood, prevention of acne, etc. It is our job as healthcare workers and researchers to come up with creative and educational ideas to ensure this knowledge gap is covered, this can be done by conducting workshops or campaigns that educate the public on this matter. Vitamin D is truly a gem of a hormone that is too often overlooked.
